# Bio-efficacy comparison of herbal-methionine and DL-methionine based on performance and blood parameters of broiler chickens

**Published:** 2014

**Authors:** Sheila Hadinia, Mahmood Shivazad, Hossein Moravej, Majid Alahyari-Shahrasb, Mohammad Mehdi Nabi

**Affiliations:** *Department of Animal Science, Agriculture and Natural Resources Pardis, University of Tehran, Karaj, Iran*

**Keywords:** Bio-efficacy, Blood parameters, Methionine, Multilinear regression

## Abstract

This study was conducted to compare the bio-efficacy of herbal methionine (H-Met) relative to DL-methionine (DL-Met) on 160 “Ross 308” broiler chickens. DL-Met and H-Met were added to the basal diet in eight experimental treatments with three and four concentrations respectively in starter, grower and finisher period. Blood parameters which were measured at 24 and 42 days of age consisted of: serum proteins (total protein, albumin and globulin), serum uric acid, serum fats (low density lipoprotein, high density lipoprotein, triglyceride and cholesterol) and serum enzymes (alanine amino transaminase and aspartate amino transaminase). Completely randomized design, multi-exponential and multilinear regressions were used to determine bio-efficacy of H-Met in terms of performance and blood parameters of broilers. The results showed that supplemented methionine (Met) sources had no significant effect on blood parameters at 24 day of age. At 42 day of age the amounts of globulin and serum high density lipoprotein (HDL) increased with supplemented Met, (*p* < 0.05). Regression analysis revealed that H-Met was 55.00, 71.00, 78.00, 47.00, 58.00 and 73.00% as efficacious as DL-Met for body weight gain, feed intake, feed conversion ratio, albumin, globulin and high density lipoprotein criteria, respectively. The average of bio-efficacy of H-Met compared to DL-Met was 67.00% and 59.00% on average across performance criteria and blood criteria respectively and was 63.00% across these two criteria tested. The results of the present study indicated that H-Met can be administered as a new and a natural source of Met in poultry industry.

## Introduction

Amino acids are considered the building blocks of proteins. Synthetic amino acids are widely used to enhance protein synthesis in animal by adding them into their diets.^1^ Synthetic methionine (Met), first limiting amino acid in broiler, can be added to many practical diets. Since rapid growth of broilers demands a high quality diet to sufficiently meet their nutrients requirements, it is necessary to supplement these diets with a Met source.^2^

The most common source of Met in poultry diets is DL-Met. This source of Met is produced by chemical synthesis from acrolein, methyl mercaptan and hydrogen cyanide.^3^ Increasing prices for petrol-derived precursors of acrolein and methyl mercaptan coupled with the increasing demand for a source of organic Met have urged producers to produce an organic source of Met called H-Met. 

Thus, it is necessary to compare this new source of Met with DL-Met in poultry nutrition. Halder and Roy examined the effect of one kind of H-Met as a source of herbal Met and DL-Met on performance of broilers and demonstrated that HerboMet can be used more efficiently than DL-Met.^4^ There are also many studies which have compared the bio-availability of methionine hydroxyl analog-free acid (MHA-FA) relative to DL-Met in broiler chickens.^5^^,^^6^ But there is lack of reports on the bio-availability of H-Met relative to DL-Met. Hence, the objective of the present experiment was to determine the bio-efficacy of H-Met compared to DL-Met based on performance and some blood parameters of broiler chickens.

## Materials and Methods

Three and four graded levels of DL-Met (98.00% purity) and H-Met (containing Met and Met + Cys 12.60 and 16.90, respectively) were added to commercial corn-soybean meal basal diets (starter 4-10 day, grower 11-24 day, and finisher 25-42 day; Table 1) in order to make up the dietary treatments (Table 2). H-Met was supplied from Arosol Company (Saharanpur, India). Constituents of H-Met included Andrographis paniculata, Ocimum sanctum, Asparagus racemosus, and Zea mays. The Met + Cys contents of the basal starter, grower and finisher diets were 0.77, 0.68 and 0.61% of DM, respectively which were below the recommended levels of Met + Cys for “Ross 308”. The addition of DL-Met and H-Met to diets provides the Met level of diets below, equivalent and excess of recommended level (Table 2). A total of 160 male 4-day-old Ross 308 were allotted to eight treatments, replicated four times with five birds per replicate. Broilers were housed in battery cages for 42 days. The feed was provided in mash form and the broilers were allowed ad libitum access to feed and water. Temperature and lighting were according to the common practice in local commercial operations. Feed intakes (FI), weight gains (WG), and feed conversion (FCR) were determined on all birds, whereas blood parameters were determined on eight birds in each treatment.

**Growth performance. **Body weight and feed intake of each replicate were measured weekly and at the end of each period and BWG, FI and FCR were calculated. 

**Blood parameters. **At days of 24 and 42 of the experimental period, 5 mL of blood was collected from wing vein from eight birds in each treatment. Blood samples were centrifuged (at 1400 *g* for 15 min) and serum was separated and then stored at –20 ˚C until further analysis. Serum samples were analyzed for total protein (TP), albumin (ALB), globulin (GLO), alanine aminotransferase (ALT), aspartate aminotransferase (AST), uric acid (UA), high density lipoprotein (HDL), low density lipoprotein (LDL), total cholesterol (CHOL) and triglyceride (TG). 

**Analysis of blood sample. **The concentration of TP was measured by Biuret method and ALB by the bromocresol green method; serum GLO was determined by subtracting serum ALB from TP value. The UA was measured by uricase method, CHOL by the cholesterol esterase-peroxidase method, HDL and LDL cholesterol, and TG using the kit package (Pars Azmoon, Tehran, Iran) and the activities of AST and ALT were determined using automatic analyzer according to the recommendation of the manufacturer.^7^

**Table 1:** Composition of starter, grower and finisher basal diets

**Ingredients (%)**	**Starter**	**Grower**	**Finisher**
**Corn**	49.86	62.30	68.50
**Soybean meal (44% Crude protein) **31.51		22.08	16.53
**Canola meal**	10.00	10.00	10.00
**Soybean oil**	3.71	1.37	0.99
**Di-Calcium phosphate**	1.94	1.62	1.49
**Oyster shell**	1.52	1.23	1.20
**Salt**	0.43	0.42	0.37
**Vitamin premix ** a	0.30	0.30	0.30
**Mineral premix ** b	0.30	0.30	0.30
**L-Lysine HCl**	0.29	0.27	0.24
**Threonine (%)**	0.14	0.11	0.08
**Calculated composition**			
**Metabolizable energy (kcal kg** ^-1^ **)**	2950	2950	3000
**Crude protein (%)**	20.94	17.95	16.08
**Calcium (%)**	1.02	0.84	0.80
**Available phosphorus (%)**	0.49	0.42	0.39
**Sodium (%)**	0.19	0.18	0.16
**Met (%)**	0.31	0.28	0.26
**Met+ Cys (%)**	0.77	0.68	0.61
**Lysine (%)**	1.24	1.03	0.88
**Threonine (%)**	0.81	0.68	0.61

a Vitamin premix provided the following per kilogram of diet: Vitamin A: 5,600 IU from all trans-retinyl acetate; Cholecalciferol: 2000 IU; Vitamin E: 20 IU from all-rac-α-tocopherol acetate; Riboflavin: 3.20 mg; Ca pantothenate: 8.00 mg; Nicotonic acid: 28.00 mg; Choline: 720 mg; Vitamin B_12_: 6.40 µg; Vitamin B_6_: 1.60 mg; Menadione: 1.60 mg (as menadione sodium bisulfate); Folic acid: 0.08 mg; D-biotin: 0.06 mg; Thiamine: 1.20 mg (as thiamine mononitrate); Ethoxyquin: 125 mg.

b Trace mineral premix provided the following in mg kg^-1^ of diet: Manganese, 40.00; Zinc, 32.00; Iron, 32.00; Copper, 3.20; Iodine, 1.20; Selenium, 0.06.

**Table 2:** Treatments and supplemented DL-Met and H-Met of the experimental diets (4-42 day).

**Groups**	**Level of supplemental Met in diet** **(% of Dry matter)**				**Difference between amounts of provided Met and required amounts of Ross’s (308) catalog** *		
	**Starter**	**Grower**	**Finisher**	**Total** ***	**Starter**	**Grower**	**Finisher**
**Control** **	–	–	–	–	–0.15	–0.11	–0.10
**DL-Met-1**	0.07	0.06	0.05	0.06	–0.08	–0.05	–0.05
**DL-Met-2**	0.15	0.11	0.10	0.11	0.00	0.00	0.00
**DL-Met-3**	0.22	0.17	0.14	0.17	+0.07	+0.06	+0.04
**H-Met-1**	0.07	0.06	0.05	0.06	–0.08	-0.05	–0.05
**H-Met-2**	0.15	0.11	0.10	0.11	0.00	0.00	0.00
**H-Met-3**	0.22	0.17	0.14	0.17	+0.07	+0.06	+0.04
**H-Met-4**	0.29	0.23	0.19	0.22	+0.14	+0.12	+0.09

* Required Met according to Ross’s (308) catalog is 0.46, 0.39 and 0.36 % for starter, grower and finisher periods respectively.

** Control = Basal diet.

*** Total = Level averages of starter, grower and finisher periods with considering the experimental days.

**Statistical** **analysis. **Data were evaluated as completely randomized designs and differences between treatment means were tested using Duncan multiple comparison test. The statistical significance was declared at a probability of *p* < 0.05. The pen mean was considered the experimental unit for all statistical analyses. A nonlinear exponential model was used to estimate the bio-efficacy of H-Met relative to DL-Met as suggested by Littell *et al*.^8^ The BWG, FCR, GLO, ALB and HDL values were analyzed by exponential regression. Simultaneous exponential regression analysis is a valid statistical means for determination of relative bio-efficacy of Met sources.^5^ The general linear model procedure using SAS (Version 9.2; SAS Institute, Carry, USA) was applied fitting the following nonlinear equation:

y= a+ b × (1- e^(c^_1_^× x^_1_^+ c^_2_^× x^_2_^)^)

where y = performance criterion, a = intercept (birds performance with basal diet), b = asymptotic response, a + b = common asymptote (maximum performance level), c_1 _= steepness coefficient for DL-Met, c_2 _= steep-ness coefficient for H-Met, and x_1_, x_2_ = dietary level of DL-Met and H-Met respectively. According to Littell* et al.,*^8^ bio-efficacy values for H-Met relative to DL-Met are given by the ratios of regression coefficient; c_2_/c_1_. The supplemented levels were confirmed by the analysis.

The FI value was analyzed by multi-linear regression as suggested by Littell *et al.*^8^ using the following equation:

y = a + (b_1_x_1_+ b_2_x_2_)

where y = performance criterion; a = performance achieved with the basal diet; b_1 _= the slope of DL-Met line; b_2 _= the slope of the H-Met^-^; x_1_, x_2_= dietary level of DL-Met and H-Met, respectively.

## Results

**Performance. **There was no mortality over the 42-day periods. The results also showed that by increasing the Met sources, BWG increased. Increasing the level of Met sources more than the required amount, resulted in a decrease in BWG (*p* < 0.05). The maximum BWG was achieved by broilers consuming the dietary treatments containing 0.11% DL-Met and 0.17% H-Met (treatments DL-Met-2 and H-Met-3). The improvement in BWG shows that the basal diet was deficient in Met (Table 3). Feed intakes also increased by the level of Met sources (*p* < 0.05). The FCR increased by addition of Met sources (*p* < 0.05). The result of the present study showed that by increasing the level of the Met sources up to 0.11% for DL-Met and 0.17% for H-Met, BWG and FI increased, but the treatments DL-Met-3 (DL-Met at 0.17%) and H-Met-4 (H-Met at 0.22%) fed broilers consumed more feed but less BWG than in treatment DL-Met-2 (DL-Met at 0.11%) and H-Met-3 (H-Met at 0.17%), resulting in increased FCR.

**Serum metabolites.** The effects of dietary treatments on blood metabolites are shown in Tables 4 - 7. No significant differences were observed among treatments at 24 day of age. At the 42 day by increasing the level of Met supplements in the diets the level of GLO and HDL increased (*p* < 0.05); and the levels of ALB decreased (*p* < 0.05). The effect of dietary treatment on TP, UA, LDL, TG, CHOL, AST and ALT were not significant at day 42. 

**Table 3:** Performance of broiler chickens fed graded levels of DL-Met and H-Met from 4 to 42 day of age

**Groups**	**Level of supplemental Met in diet (% of DM)**	**BWG** **(g)**	**FI** **(g)**	**FCR**
**Control** *	-	2132.67^d^	3720.11^d^	1.74^b^
**DL-Met-1**	0.06	2356.93^c^	4131.88^c^	1.75^b^
**DL-Met-2**	0.11	2490.75^a^	4394.76^b^	1.76^b^
**DL-Met-3**	0.17	2465.62^b^	4643.48^a^	1.88^a^
**H-Met-1**	0.06	2245.49^d^	3736.91^d^	1.66^c^
**H-Met-2**	0.11	2352.47^c^	4146.54^c^	1.76^b^
**H-Met-3**	0.17	2476.45^a^^b^	4407.25^b^	1.78^b^
**H-Met-4**	0.22	2463.87^b^	4686.31^a^	1.90^a^
**SEM**	-	8.17	22.85	0.02

* Control= Basal diet.

a-d Mean values in a column with no common superscript differ significantly (*p* < 0.05).

**Table 4:** Effects of graded levels of Met sources on serum bio-chemical parameters of broiler chickens at 24 day of age

**Groups**	**TP** **(g dL** ^-1^ **)**	**ALB** **(g dL** ^-1^ **)**	**GLO** **(g dL** ^-1^ **)**	**AST** **(IU L** ^-1^ **)**	**ALT** **(IU L** ^-1^ **)**	**UA** **(mg dL** ^-1^ **)**
**Control** *	2.53	1.31	1.22	228.13	36.60	4.25
**DL-Met-1**	2.53	1.29	1.24	228.05	36.43	4.26
**DL-Met-2**	2.53	1.28	1.25	226.00	36.23	4.38
**DL-Met-3**	2.53	1.26	1.27	228.09	36.47	4.33
**H-Met-1**	2.56	1.31	1.26	226.50	36.36	4.32
**H-Met-2**	2.55	1.29	1.26	227.85	36.27	4.28
**H-Met-3**	2.52	1.29	1.24	226.00	36.25	4.41
**H-Met-4**	2.57	1.27	1.30	228.08	36.45	4.42
**SEM**	0.09	0.08	0.11	0.71	0.16	0.16

TP = Total protein; ALB = albumin; GLO = globulin; AST = aspartate aminotransferase; ALT = alanine aminotransferase; UA = uric acid;

* Control= Basal diet.

**Table 5:** Effects of graded levels of Met sources on serum bio-chemical parameters (mg dL^-1^) of broiler chickens at 24 day of age

**Groups**	**CHOL**	**TG**	**HDL**	**LDL**
**Control** *	78.55	30.30	51.64	19.02
**DL-Met-1**	79.08	29.81	51.75	19.03
**DL-Met-2**	79.20	29.88	52.00	19.36
**DL-Met-3**	78.56	30.79	52.45	19.30
**H-Met-1**	78.80	29.78	51.68	19.23
**H-Met-2**	79.18	29.88	51.70	19.03
**H-Met-3**	79.25	29.78	51.85	19.06
**H-Met-4**	79.23	30.76	52.00	19.35
**SEM**	0.33	0.39	0.29	0.14

CHOL = cholesterol; TG = triglyceride; HDL = high density lipoprotein; LDL = low density lipoprotein;

* Control = Basal diet.

**Table 6:** Effects of graded levels of Met sources on serum biochemical parameters of broiler chickens at 42 day of age

**Groups**	**TP** **(g dL** ^-1^ **)**	**ALB** **(g dL** ^-1^ **)**	**GLO** **(g dL** ^-1^ **)**	**AST** **(IU L** ^-1^ **)**	**ALT** **(IU L** ^-1^ **)**	**UA** **(mg dL** ^-1^ **)**
**Control** *	3.33	1.87^a^	1.46^d^	234.20	39.00	10.33
**DL-Met-1**	3.33	1.68^b^	1.66^c^	231.67	38.20	10.41
**DL-Met-2**	3.33	1.62^c^^d^	1.71^b^^c^	230.11	37.80	10.43
**DL-Met-3**	3.35	1.59^d^	1.76^a^^b^	233.75	38.64	10.45
**H-Met-1**	3.32	1.86^a^	1.46^d^	231.50	38.50	10.36
**H-Met-2**	3.34	1.69^b^	1.66^c^	231.00	38.38	10.38
**H-Met-3**	3.33	1.65b^c^	1.68^c^	230.80	37.98	10.42
**H-Met-4**	3.38	1.60^d^	1.79^a^	234.17	38.79	10.45
**SEM**	0.02	0.07	0.09	1.38	0.43	0.12

a–d Means values within a column without common superscripts differ statistically (*p *< 0.05);

* Control= Basal diet.

**Table 7:** Effects of graded levels of Met sources on serum bio-chemical parameters (mg dL^-1^) of broiler chickens at 42 day of age

**Groups**	**CHOL**	**TG**	**HDL**	**LDL**
**Control** *	81.78	33.32	53.51^c^	19.98
**DL-Met-1**	84.23	33.46	55.03^c^	21.06
**DL-Met-2**	86.95	33.47	64.35^b^	21.30
**DL-Met-3**	88.20	33.48	66.51^a^	22.14
**H-Met-1**	84.08	33.43	55.03^c^	20.10
**H-Met-2**	84.66	33.46	55.05^c^	21.10
**H-Met-3**	87.22	33.48	64.53^b^	21.37
**H-Met-4**	88.45	33.50	66.61^a^	22.50
**SEM**	2.97	0.14	0.65	0.91

a–c Means values within a column without common superscripts differ statistically (*p* < 0.05);

* Control= Basal diet.

**Table 8:** Estimated effectiveness of H-Met relative to DL-Met based on BWG, FI, FCR and blood parameters of broiler chickens

**Variable**	**Performance**					**Blood parameters**		
	**BWG**		**FI**	**FCR**		**ALB**	**GLO**	**HDL**
**Bio-efficacy**	55		71	78		47	58	73
**Mean**	67					59		
**Total mean**		63						

**Fig. 1:**
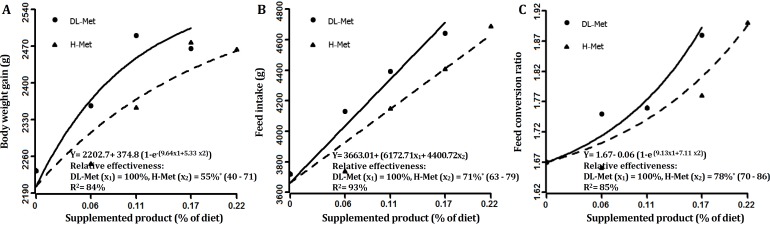
Bio-efficacy H-Met relative to DL-Met using **A)** body weight gain, **B)** feed intake, and **C)** feed conversion ratio in male Ross 308 broilers (4-42 days of age). Zero level indicates control. Values in parentheses indicate the 95% confidence interval

**Fig. 2:**
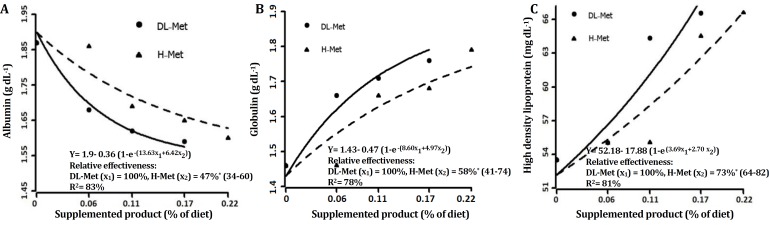
Bio-efficacy H-Met relative to DL-Met using **A)** albumin, **B)** globulin, and **C)** high density lipoprotein in male Ross 308 broilers (4-42 days of age). Zero level indicates control. Values in parentheses indicate the 95% confidence interval.

Relative effectiveness of H-Met was significantly lower than that of DL-Met, (Figs. 1 and 2 for details).

## Discussion

Bunchasak and Keawarun found that Met deﬁciencies depressed the FI of broiler chicks due to amino acid imbalances.^9^ It can be assumed that, under amino acid imbalances, chicks lose the potential to adjust FI to satisfy their amino acid requirements; the main positive effect of Met supplementation may come from its improvement of FI via the amino acid balance.^10^ As Met plays an important role in protein synthesis, in low amounts of Met, protein synthesis and cysteine biosynthesis from Met will reduce. Thus, it cannot play its key role for synthesizing proteins. Salmon showed the consumption of a disproportionate amount of Met impaired growth and caused tissue damage.^11^ Theories representing the reasons are: 1) The depletion of hepatic ATP in adenylating Met to S-adenosyl Met (SAM);^12^ 2) Depletion of methyl acceptors in the conversion of SAM to S-adenosyl-homocysteine;^13^ 3) Metabolism of the labile methyl group of Met via an alternate pathway not requiring formation of SAM is the means by which the toxicity is exerted.^14^ The normal trans-sulfuration pathway of Met catabolism involves formation of homocysteine (HCY), which donates its sulfur moiety to serine to eventually form 1 mole of cysteine per mole of Met catabolized.^15^ The result of growth performance in the current study did not confirm the result of Halder and Roy who reported that there are no significant differences with utilization H-Met in comparison with DL-Met in the same level.^4^ In fact, the results of present study showed significant differences between the same levels of either DL-Met or H-Met. These findings are in agreement with the observation of Xie *et al. *who reported an increase and a subsequent decrease in BWG as dietary Met increased.^16^ Therefore, it seems that supplemented Met sources more than the required amount for broilers do not improve the BWG. Han and Baker indicated that 0.50% excess of Met is not harmful to young broiler chicks fed corn-soybean meal diet.^15^ As Met supplementation levels increased regardless of the sources, FI was significantly increased and FCR was also increased due to the higher FI in higher Met supplemented diets.

Broilers with higher body weight gains showed a higher concentration of serum total protein compared to the lighter broilers possibly due to higher demand for lean tissue maintenance and turnover.^17^ Feeding low protein and amino acid diets seemed to be associated with a decrease in serum TP and serum ALB in chickens.^18^ ALB is a blood transport protein which binds many biomolecules and drugs including hormones, lipoproteins and amino acids.^19^^,^^20^

Smith suggested that ALB as a major protein in the blood of laying hens is decomposed with an increase in the requirement of amino acids leading to a decrease in its concentration.^21^^,^^22^ One of the reasons for ALB reduction in the current study can be related to its decomposition for supplying amino acids requirements or increasing GLO level. In contrast, Hind *et al.* showed that the level of Met did not have any effect on serum TP, ALB and GLO, though a numerical increase was observed in the amount of GLO.^23^

Among the amino acids, total sulfur amino acids (TSAA) have the highest potential for adjustment of fat metabolism.^24^ The results showed that serum HDL increased by addition of Met sources to the diets. Met-supplementation increased HDL and apolipoprotein A-I (apo A-I) in blood.^25^ Moreover, hepatic mRNA levels and transcription rates of apo A-I gene increased by the addition of Met to soy protein.^25^ Therefore, the stimulation of apo A-I gene by Met might be responsible for increasing HDL resulted from the addition of Met to the diet.^28^ It seems that HDL is elevated by sulfur amino acids (SAAs) through an increase in apo A-I gene expression in the liver.^26^ Taurine, one of SAAs, is synthesized mainly in the liver as an end product of SAAs catabolism and HDL tends to be elevated by dietary taurine.^27^ Taurine exerts its effect through post-translational modiﬁcation of regulatory proteins such as those associated with phosphorylation/ dephosphorylation or ligand binding to nuclear receptors.^26^ Reportedly, Met could cause alteration in lipogenesis and lipolysis in broiler chicks.^28^ This finding indicates that supplementation of Met facilitates efficient lipid metabolism in the liver and its transportation to the tissues and consequently it may reduce the incidence of fatty liver in birds as Halder and Roy reported for Herbo-Met.^4^

Hoehler *et al*. demonstrated that the design of the trial can be done either equimolar or weight to weight comparison of the two Met sources, although the results are not exactly the same but both of these methods are true.^29^ In the present study the addition of each Met sources was done on a weight basis.

There are several hypotheses regarding why H-Met has a lower bio-efficacy relative to DL-Met. There are some possible explanations for lower bio-efficacy of H-Met relative to DL-Met as Hoehler *et al.* and Payne *et al.* explained in their studies.^5^^,^^6^ The poor utilization of the polymeric forms of H-Met relative to DL-Met polymeric form may be one of the main reasons for its lower bio-efficacy. 

Considering the results of the present study, it seems that H-Met is a suitable natural substitute for DL-Met on the broilers diet, if the cost is suitable. As the results showed, the level of 0.17% from H-Met did not have a significant effect with the level of 0.11% from DL-Met and these two levels of Met sources could obtain the maximum BWG. Therefore, it can be calculated that H-Met should consume 1.55 more times than DL-Met to obtain the same response. Hence, the cost of H-Met should be 45.00% of that of DL-Met in order to be economical. In conclusion, on average bio-efficacy was 63.00% for H-Met based on growth performance and blood parameters. Although, the relative effectiveness of H-Met was significantly lower than DL-Met in broiler chickens, H-Met can be administered as a new and a natural source of Met in poultry industry. Also, as it is stated in above paragraph the cost of H-Met should be considered 45.00% of DL-Met cost to be economical.
